# Supplementary material for: Effects of a Lacto-Ovo-Vegetarian Diet on the Plasma Lipidome and Its Association with Atherosclerotic Burden in Patients with Coronary Artery Disease—A Randomized, Open-Label, Cross-over Study
Source: Nutrients. 2020 Nov 23;12(11):3586. doi: 10.3390/nu12113586 (PMC7700669; doi:10.3390/nu12113586)
Supplement: Supplementary file 1 [file nutrients-12-03586-s001.pdf]

SUPPLEMENTARY METHODS

The serum samples were randomized and extracted using a modified version of the Folch procedure. Shortly, 10 µL of 0.9% NaCl and 120 µL of CHCl3: MeOH (2:1, v/v) containing 2.5 µg mL-1 internal standards solution (for quality control and normalisation purposes) were added to 10 µL of each plasma sample. The standard solution contained the following compounds: 1,2-diheptadecanoyl-sn-glycero-3-phosphoethanolamine (PE(17:0/17:0)), N-heptadecanoyl-D-erythro-sphingosylphosphorylcholine (SM(d18:1/17:0)), N-heptadecanoyl-D-erythro-sphingosine (Cer(d18:1/17:0)), 1,2-diheptadecanoyl-sn-glycero-3-phosphocholine (PC(17:0/17:0)), 1-heptadecanoyl-2-hydroxy-sn-glycero-3-phosphocholine (LPC(17:0)) and 1-palmitoyl-d31-2-oleoyl-sn-glycero-3-phosphocholine (PC(16:0/d31/18:1)), 1-palmitoyl-d31-2-oleoyl-sn-glycero-3-phosphocholine (PC (16.0/d31/18:1) were purchased from Avanti Polar Lipids, Inc. (Alabaster, AL, USA), tripalmitin- Triheptadecanoylglycerol (TG(17:0/17:0/17:0)) (Larodan AB, Solna, Sweden). The samples were vortex mixed and incubated on ice for 30 min after which they were centrifuged (9400 × g, 3 min, 4 °C). 60 µL from the lower layer of each sample was then transferred to a glass vial with an insert and 60 µL of CHCl3: MeOH (2:1, v/v) was added to each sample. The samples were re-randomized and stored at -80 °C until analysis.

Calibration curves using 1-hexadecyl-2-(9Z-octadecenoyl)-sn-glycero-3-phosphocholine (PC(16:0/18:1(9Z))), 1-(1Z-octadecenyl)-2-(9Z-octadecenoyl)-sn-glycero-3-phosphocholine (PC(16:0/16:0)), 1-octadecanoyl-sn-glycero-3-phosphocholine (LPC(18:0)), (LPC18:1), PE (16:0/18:1), (2-aminoethoxy)[(2R)-3-hydroxy-2-[(11Z)-octadec-11-enoyloxy]propoxy]phosphinic acid (LysoPE(18:1)), N-(9Z-octadecenoyl)-sphinganine (Cer (d18:0/18:1(9Z))), 1-hexadecyl-2-(9Z-octadecenoyl)-sn-glycero-3-phosphoethanolamine (PE (16:0/18:1)) from Avanti Polar Lipids, Inc., 1-Palmitoyl-2-

Hydroxy-sn-Glycero-3-Phosphatidylcholine (LPC(16:0)) and 1,2,3 trihexadecanoalglycerol (TG16:0/16:0/16:0), 1,2,3-trioctadecanoylglycerol (TG(18:0/18:0/18:0)) and ChoE(18:0), 3β-Hydroxy-5-cholestene 3-linoleate (ChoE(18:2)) from from Larodan, were prepared prepared to the following concentration levels: 100, 500, 1000, 1500, 2000 and 2500 ng mL−1 (in CHCl3:MeOH, 2:1, v/v) including 1000 ng mL-1 of each internal standard.

The samples were analyzed using an ultra-high-performance liquid chromatography quadrupole time-of-flight mass spectrometry method (UHPLC-Q-TOF-MS). Briefly, the UHPLC system used in this work was a 1290 Infinity system from Agilent Technologies (Santa Clara, CA, USA). The system was equipped with a multi sampler (maintained at 10 °C), a quaternary solvent manager and a column thermostat (maintained at 50 °C). Separations were performed on an ACQUITY UPLC® BEH C18 column (2.1 mm × 100 mm, particle size 1.7 µm) by Waters (Milford, USA). The mass spectrometer coupled to the UHPLC was a 6545 quadrupole time of flight (Q-TOF) from Agilent Technologies interfaced with a dual jet stream electrospray (dual ESI) ion source. All analyses were performed in positive ion mode and MassHunter B.06.01 (Agilent Technologies) was used for all data acquisition.

MS data processing was performed using open source software MZmine 2.34. The following steps were applied in the processing: 1.) Mass detection with a noise level of 750 2.) Chromatogram builder with a min time span of 0.08 min, min height of 1000 and a m/z tolerance of 0.006 m/z or 10.0 ppm, 3.) Chromatogram deconvolution using the local minimum search algorithm with a 70% chromatographic threshold, 0.05 min minimum RT range, 5% minimum relative height, 1000 minimum absolute height, and a peak duration range of 0.08 - 2.0, 4.), Isotopic peak grouper with a m/z tolerance of 5.0 ppm, RT tolerance

of 0.05 min, maximum charge of 2 and with the most intense isotope set as the representative isotope, 5.) Join aligner with a m/z tolerance of 0.006 009 or 10.0 ppm and a weight for of 2, a RT tolerance of 0.1 min and a weight of 1 and with no requirement of charge state or ID and no comparison of isotope pattern, 6.) Peak list row filter with a minimum of 10% of the sample), 7.) Gap filling using the same RT and m/z range gap filler algorithm with an m/z tolerance of 0.006 009 m/z or 1011.0 ppm, 7.) Identification of lipids using a custom database search with an m/z tolerance of 0.006 009 m/z or 10.0 ppm and a RT tolerance of 0.1 min, 8.) Normalization using internal standards (PE (17:0/17:0), SM (d18:1/17:0), Cer (d18:1/17:0), LPC (17:0), TG (1617:0/1617:0/1617:0)-13C3 and PC (16:0/d30/18:1)) for identified lipids and closest ISTD for the unknown lipids. The lipid concentrations were calculated using lipid-class specific calibration curves.

Quality control was performed throughout the dataset by including blanks, pure standard samples, extracted standard samples and pooled control plasma samples. Relative standard deviations (%RSDs) for internal standards in all samples was on average 25.4% (raw variation) and the RSD% for the identified lipids in the pooled samples (n = 5) was on average 15.6 %.

Table S1. Baseline characteristics of study participants in VERDI trial by number of stenotic coronary arteries and longitudinal atherosclerotic extent prior to percutanous coronary intervention.

Clinical parameters	SCA <4 (n = 16)	SCA ≥4 (n = 14)	SES <18 (n = 16)	SES ≥18 (n = 14)
Age	66±10	65±8	63±10	67±5*
Male sex, n (%)	15 (94)	13 (93)	16 (100)	12 (86)
Weight kg	86±7	83±15	88±9	80 ±12*
Oxidized LDL-C, mmol/L	40±11	43±12	39±11	44±12
Total cholesterol, mmol/L	3.5±0.6	3.4±0.6	3.5±0.7	3.5±0.5
LDL-C, mmol/L	1.7±0.4	1.6±0.5	1.6±0.4	1.6±0.5

HDL-C, mmol/L	1.2±0.3	1.3±0.3	1.2±0.3	1.3±0.3
Triacylglycerols,mmol/L	1.1±0.3	1.0±0.4	1.1±0.4	1.0±0.3
Apolipoprotein B, g/L	0.7±0.1	0.6±0.1	0.7±0.1	0.6±0.1
Apolipoprotein A1, g/L	1.4±0.2	1.4±0.2	1.4±0.2	1.4±0.2
ApoB/ApoA1 ratio	0.5±0.1	0.5 ± 0.1	0.5±0.1	0.5±0.1
Hemoglobin A1c, mmol/mol	40.0±4.3	36.7±3.0*	39.4±5	37.4±3
High sensitivity c-reactive protein, mg/L	1.3±1.6	1.4±1.6	1.5±1.6	1.1±1.6
Systolic bp, mm Hg	138±13	141±22	135±15	145±19
Diastolic bp, mm Hg	87±8	87±12	86±6	88±13
Heart rate, bpm	64±7	67±11	64±9	67±10

Data are shown as n (%) or mean± standard deviation.*$P < 0.05$. Bp: blood pressure; HDL: High-density lipoprotein; LDL: Low-density lipoprotein; SCA: Stenotic coronary arteries; SES: Sullivan extent score.

Table S2. Plasma lipidomic identification by lipid class and the clustering information obtained using the weighted gene correlation network analysis.

**Lipids**	**Class**	**Modules**
Cer(d18:1/16:0)	Ceramides	turquoise
Cer(d40:1)/(d18:1/22:0)	Ceramides	black
Cer(d18:1/22:0)	Ceramides	black
Cer(d18:1/23:0)	Ceramides	black
Cer(d18:1/24:0)	Ceramides	black
Cer(d18:1/24:1)	Ceramides	black
Cer(d18:1/25:0)	Ceramides	black
Cer(d42:1)	Ceramides	black
CE(16:0)	Cholesteryl ester	grey
CE(20:5)	Cholesteryl ester	grey
CE(16:1)	Cholesteryl ester	grey
CE(18:0)	Cholesteryl ester	turquoise
CE(18:1)	Cholesteryl ester	purple
CE(18:2)	Cholesteryl ester	purple
CE(20:4)	Cholesteryl ester	purple
DG(36:3)	Diacylglycerides	blue
PC(O-42:5)/(o-22:1/20:4)	Ether phosphatidylcholines	grey
PC(O-38:4)_2	Ether phosphatidylcholines	grey
PC(O-40:5)#	Ether phosphatidylcholines	grey
PC(O-32:0)	Ether phosphatidylcholines	turquoise
PC(O-32:1)	Ether phosphatidylcholines	turquoise

PC(O-34:2)	Ether phosphatidylcholines	turquoise
PC(O-34:3)	Ether phosphatidylcholines	turquoise
PC(O-34:3)NH4adduct	Ether phosphatidylcholines	turquoise
PC(O-36:3)	Ether phosphatidylcholines	turquoise
PC(O-36:3)#	Ether phosphatidylcholines	turquoise
PC(O-36:4)	Ether phosphatidylcholines	turquoise
PC(O-36:5)	Ether phosphatidylcholines	turquoise
PC(O-38:4)	Ether phosphatidylcholines	turquoise
PC(O-38:4)#	Ether phosphatidylcholines	turquoise
PC(O-38:5)	Ether phosphatidylcholines	turquoise
PC(O-38:5)#	Ether phosphatidylcholines	turquoise
PC(O-38:6)	Ether phosphatidylcholines	turquoise
PC(O-40:5)	Ether phosphatidylcholines	turquoise
PC(O-38:6)#	Ether phosphatidylcholines	magenta
HexCer(d18:1/22:0)	Hexosylceramides	turquoise
HexCer(d18:1/24:0)	Hexosylceramides	black
LacCer(d18:1/16:0)	Lactosylceramides	turquoise
LPC(16:0)	lyso-phosphatidylcholines	grey
LPC(16:0e)	lyso-phosphatidylcholines	grey
LPC(16:0p)	lyso-phosphatidylcholines	grey
LPC(14:0)	lyso-phosphatidylcholines	brown
LPC(18:1)	lyso-phosphatidylcholines	yellow
LPC(18:0)	lyso-phosphatidylcholines	black
LPC(15:0)	lyso-phosphatidylcholines	pink
PC(44:5)/(22:2/22:3)	Phosphatidylcholines	grey
PC(36:5)	Phosphatidylcholines	grey
PC(36:4)/(18:3/18:1)	Phosphatidylcholines	turquoise
PC(37:3)/(18:3/19:0)	Phosphatidylcholines	turquoise
PC(38:1)	Phosphatidylcholines	turquoise
PC(38:2)/(14:1/24:1)	Phosphatidylcholines	turquoise
PC(39:0)/(13:0/26:0)	Phosphatidylcholines	turquoise
PC(42:8)/(20:3/22:5)	Phosphatidylcholines	turquoise
PC(42:8)/(20:3/22:5)#	Phosphatidylcholines	turquoise
PC(16:0e/18:1(9Z))	Phosphatidylcholines	turquoise
PC(18:0p/18:1(9Z))	Phosphatidylcholines	turquoise
PC(18:0p/22:6)	Phosphatidylcholines	turquoise
PC(35:2)	Phosphatidylcholines	turquoise

PC(35:3)	Phosphatidylcholines	turquoise
PC(36:2)#	Phosphatidylcholines	turquoise
PC(36:3)	Phosphatidylcholines	turquoise
PC(36:3)#	Phosphatidylcholines	turquoise
PC(36:4)_3	Phosphatidylcholines	turquoise
PC(37:2)	Phosphatidylcholines	turquoise
PC(38:3)	Phosphatidylcholines	turquoise
PC(38:4)#	Phosphatidylcholines	turquoise
PC(40:5)#	Phosphatidylcholines	turquoise
PC(P-18:0/22:6)	Phosphatidylcholines	turquoise
PC(38:6)	Phosphatidylcholines	blue
PC(39:6)	Phosphatidylcholines	blue
PC(40:6)	Phosphatidylcholines	blue
PC(40:7)	Phosphatidylcholines	blue
PC(37:5)	Phosphatidylcholines	brown
PC(16:0/16:0)	Phosphatidylcholines	yellow
PC(16:0/18:1)	Phosphatidylcholines	yellow
PC(30:0)	Phosphatidylcholines	yellow
PC(31:0)	Phosphatidylcholines	yellow
PC(32:1)	Phosphatidylcholines	yellow
PC(32:2)	Phosphatidylcholines	yellow
PC(33:0)	Phosphatidylcholines	yellow
PC(33:1)	Phosphatidylcholines	yellow
PC(34:2)	Phosphatidylcholines	yellow
PC(34:3)	Phosphatidylcholines	yellow
PC(35:1)	Phosphatidylcholines	yellow
PC(36:1)	Phosphatidylcholines	yellow
PC(36:2)	Phosphatidylcholines	yellow
PC(36:4)	Phosphatidylcholines	yellow
PC(36:4)#	Phosphatidylcholines	yellow
PC(36:5)#	Phosphatidylcholines	yellow
PC(38:6)#	Phosphatidylcholines	yellow
PC(40:8)	Phosphatidylcholines	yellow
PC(37:3)	Phosphatidylcholines	pink
PC(38:5)	Phosphatidylcholines	pink
PC(40:4)	Phosphatidylcholines	pink
PC(40:5)	Phosphatidylcholines	pink

PC(40:6)#	Phosphatidylcholines	pink
PC(35:4)	Phosphatidylcholines	tan
PC(36:4)_2	Phosphatidylcholines	tan
PC(37:4)	Phosphatidylcholines	tan
PC(38:4)	Phosphatidylcholines	tan
PE(O-38:5)orPE(P-38:4)	Phosphatidylethanolamine	turquoise
PE(P-18:0/22:6)	Phosphatidylethanolamine	yellow
PE(16:0/18:1)	Phosphatidylethanolamine	greenyellow
PE(34:2)	Phosphatidylethanolamine	greenyellow
PE(36:4)	Phosphatidylethanolamine	greenyellow
PE(38:4)	Phosphatidylethanolamine	greenyellow
PE(38:6)	Phosphatidylethanolamine	greenyellow
PG(O-39:0)/(O-20:0/19:0)	Phosphatidylglycerol	turquoise
PG(O-41:0)/(O-20:0/21:0)	Phosphatidylglycerol	turquoise
PI(44:4)	Phosphatidylinositol	turquoise
PI(38:7)	Phosphatidylinositol	pink
PI(18:0/20:4)	Phosphatidylinositol	pink
SM(39:2)	Sphingomyelins	turquoise
SM(37:1)	Sphingomyelins	turquoise
SM(40:2)	Sphingomyelins	turquoise
SM(d39:1)	Sphingomyelins	turquoise
SM(d42:3)	Sphingomyelins	turquoise
SM(d34:2)	Sphingomyelins	turquoise
SM(d18:1/12:0)	Sphingomyelins	turquoise
SM(d18:1/24:0)	Sphingomyelins	turquoise
SM(d18:2/14:0)	Sphingomyelins	turquoise
SM(d32:1)	Sphingomyelins	turquoise
SM(d33:1)	Sphingomyelins	turquoise
SM(d34:1)	Sphingomyelins	turquoise
SM(d36:1)	Sphingomyelins	turquoise
SM(d36:2)	Sphingomyelins	turquoise
SM(d38:2)	Sphingomyelins	turquoise
SM(d40:1)	Sphingomyelins	turquoise
SM(d41:1)	Sphingomyelins	turquoise
SM(d41:2)	Sphingomyelins	turquoise
SM(d42:2)	Sphingomyelins	turquoise
SM(d18:0/16:0)	Sphingomyelins	purple

SM(d36:0)	Sphingomyelins	purple
SM(d35:1)	Sphingomyelins	tan
TG(56:5)	Triacylglycerol	grey
TG(53:3)	Triacylglycerol	blue
TG(14:0/18:2/18:2)	Triacylglycerol	blue
TG(16:0/18:2/18:2)	Triacylglycerol	blue
TG(16:0/18:2/18:3)	Triacylglycerol	blue
TG(16:0/18:2/18:3)#	Triacylglycerol	blue
TG(18:1/18:1/16:0)	Triacylglycerol	blue
TG(18:2/18:1/16:0)	Triacylglycerol	blue
TG(18:2/18:1/18:1)#	Triacylglycerol	blue
TG(18:2/18:2/18:2)orTG(18:3/18:2/18:1)	Triacylglycerol	blue
TG(48:3)	Triacylglycerol	blue
TG(50:0)	Triacylglycerol	blue
TG(50:1)#	Triacylglycerol	blue
TG(50:3)	Triacylglycerol	blue
TG(50:3)#	Triacylglycerol	blue
TG(50:5)	Triacylglycerol	blue
TG(51:2)	Triacylglycerol	blue
TG(51:2)_2	Triacylglycerol	blue
TG(51:3)	Triacylglycerol	blue
TG(51:4)	Triacylglycerol	blue
TG(52:2)	Triacylglycerol	blue
TG(52:3)	Triacylglycerol	blue
TG(52:4)	Triacylglycerol	blue
TG(52:5)	Triacylglycerol	blue
TG(53:2)	Triacylglycerol	blue
TG(53:3)	Triacylglycerol	blue
TG(53:4)	Triacylglycerol	blue
TG(53:5)	Triacylglycerol	blue
TG(55:5)	Triacylglycerol	blue
TG(46:0)/(14:0/16:0/16:0)	Triacylglycerol	brown
TG(46:2)/(16:1/14:0/16:1)	Triacylglycerol	brown
TG(48:2)/(14:1/16:1/18:0)	Triacylglycerol	brown
TG(49:3)/(15:0/18:2/16:1)	Triacylglycerol	brown
TG(52:0)	Triacylglycerol	brown
TG(14:0/16:0/18:1)	Triacylglycerol	brown

TG(14:0/18:1/18:1)	Triacylglycerol	brown
TG(16:0/16:0/12:0)	Triacylglycerol	brown
TG(16:1/18:1/12:0)	Triacylglycerol	brown
TG(18:1/12:0/18:1)orTG(18:2/16:0/14:0)	Triacylglycerol	brown
TG(47:0)	Triacylglycerol	brown
TG(47:1)	Triacylglycerol	brown
TG(48:1)	Triacylglycerol	brown
TG(49:0)	Triacylglycerol	brown
TG(49:0)#	Triacylglycerol	brown
TG(49:1)	Triacylglycerol	brown
TG(49:2)	Triacylglycerol	brown
TG(50:1)	Triacylglycerol	brown
TG(50:2)	Triacylglycerol	brown
TG(51:1)	Triacylglycerol	brown
TG(51:2)#	Triacylglycerol	brown
TG(16:0/18:2/22:6)	Triacylglycerol	green
TG(16:0/22:5/18:1)orTG(20:4/18:1/18:1)	Triacylglycerol	green
TG(18:0/18:1/20:4)	Triacylglycerol	green
TG(18:1/18:2/18:2)	Triacylglycerol	green
TG(18:2/22:5/16:0)	Triacylglycerol	green
TG(52:6)	Triacylglycerol	green
TG(54:6)	Triacylglycerol	green
TG(54:7)	Triacylglycerol	green
TG(54:7)#	Triacylglycerol	green
TG(56:6)	Triacylglycerol	green
TG(58:6)	Triacylglycerol	green
TG(58:9)	Triacylglycerol	green
TG(56:3)	Triacylglycerol	red
TG(56:4)	Triacylglycerol	red
TG(18:1/18:1/18:1)	Triacylglycerol	red
TG(18:2/18:1/18:1)	Triacylglycerol	red
TG(54:3)	Triacylglycerol	red
TG(54:4)	Triacylglycerol	red
TG(54:5)	Triacylglycerol	red
TG(56:3)	Triacylglycerol	red
TG(56:4)	Triacylglycerol	red
TG(60:7)	Triacylglycerol	red

TG(48:4)	Triacylglycerol	pink
TG(54:1)	Triacylglycerol	magenta
TG(55:1)	Triacylglycerol	magenta
TG(16:0/16:0/16:0)	Triacylglycerol	magenta
TG(16:0/18:0/18:1)	Triacylglycerol	magenta
TG(48:0)	Triacylglycerol	magenta
TG(50:0)#	Triacylglycerol	magenta
TG(54:1)	Triacylglycerol	magenta

Cer: ceramide; CE: cholesteryl ester; DAG: diacylglyceride; HexCer: Hexosylceramide; Hs-CRP: high-sensitive c-reactive protein; LacCer: lactosylceramide; LPC: lyso-phosphatidylcholine; O-PC: ether phosphatidylcholine; PC: phosphatidylcholine; PE: phosphatidylethanolamine; PG: phosphatidylglycerol, PI: phosphatidylinositol; SM: sphingomyelin; TG: Triacylglycerol. # Lipids with same fatty acyl chains (Cx:y) were presented in the dataset, where x represents the number of carbon atoms and y the number of double bonds of the fatty acyl chain.

Table S3. Effects of diets on plasma lipidome.

Lipids	Baseline		Vegetarian diet		Meat diet		Baseline vs. vegetarian diet		Baseline vs. meat diet		Vegetarian diet vs. meat diet	
	Least square means	SE	Least square means	SE	Least square means	SE	*P*	FDR p	*P*	FDR p	*P*	FDR p
CE(16:0)	6.36	0.72	7.67	0.72	7.86	0.71	0.20	0.37	0.15	0.51	0.00	0.00
CE(16:1)	5.15	0.09	5.17	0.09	5.12	0.08	0.86	0.92	0.72	0.96	0.00	0.00
CE(18:0)	5.10	0.08	4.72	0.08	5.02	0.09	0.00	0.00	0.33	0.74	0.00	0.00
CE(18:1)	9.17	0.02	9.12	0.02	9.13	0.02	0.03	0.08	0.04	0.27	0.00	0.00
CE(18:2)	9.33	0.02	9.30	0.02	9.28	0.02	0.24	0.42	0.10	0.44	0.00	0.00
CE(20:4)	8.76	0.04	8.68	0.04	8.73	0.03	0.02	0.05	0.24	0.63	0.00	0.00
CE(20:5)	7.49	0.06	7.23	0.06	7.24	0.06	0.00	0.01	0.00	0.02	0.00	0.00
Cer(d18:1/16:0)	2.15	0.08	1.66	0.08	1.95	0.06	0.00	0.00	0.03	0.25	0.00	0.00
Cer(d18:1/22:0)	2.92	0.06	2.79	0.06	2.81	0.05	0.06	0.15	0.08	0.40	0.00	0.00
Cer(d18:1/23:0)	3.05	0.06	3.00	0.06	2.95	0.09	0.48	0.63	0.38	0.80	0.00	0.00
Cer(d18:1/24:0)	4.22	0.07	4.08	0.07	4.15	0.07	0.12	0.27	0.47	0.87	0.00	0.00
Cer(d18:1/24:1)	3.59	0.06	3.44	0.06	3.50	0.06	0.02	0.06	0.22	0.59	0.00	0.00
Cer(d18:1/25:0)	2.50	0.13	2.61	0.13	2.52	0.12	0.49	0.63	0.90	1.00	0.00	0.00
Cer(d40:1)/(d18:1/22:0)	3.09	0.06	2.90	0.06	3.00	0.06	0.01	0.03	0.21	0.59	0.00	0.00
Cer(d42:1)	4.35	0.06	4.23	0.06	4.26	0.07	0.10	0.24	0.31	0.72	0.00	0.00
DAG(36:3)	1.51	0.08	1.60	0.08	1.35	0.10	0.44	0.61	0.14	0.49	0.00	0.01
HexCer(d18:1/22:0)	2.21	0.08	2.02	0.08	2.22	0.07	0.01	0.04	0.78	0.96	0.00	0.01
HexCer(d18:1/24:0)	2.53	0.07	2.36	0.07	2.42	0.08	0.05	0.14	0.21	0.59	0.00	0.01

LacCer(d18:1/16:0)	3.84	0.05	3.65	0.05	3.71	0.05	0.00	0.00	0.01	0.13	0.00	0.01
LPC(16:0)	6.81	0.05	6.74	0.05	6.77	0.05	0.09	0.21	0.53	0.94	0.00	0.01
LPC(16:0e)	1.49	0.09	1.18	0.09	1.49	0.08	0.00	0.02	0.94	1.00	0.00	0.01
LPC(16:0p)	1.73	0.06	1.64	0.06	1.78	0.07	0.32	0.49	0.55	0.94	0.00	0.01
LPC(18:0)	5.59	0.06	5.48	0.06	5.56	0.05	0.03	0.08	0.58	0.94	0.00	0.01
LPC(18:1)	5.72	0.05	5.67	0.05	5.73	0.05	0.26	0.44	0.69	0.96	0.00	0.01
LysoPC(14:0)	2.36	0.08	2.36	0.08	2.38	0.08	0.96	0.98	0.86	0.98	0.00	0.01
LysoPC(15:0)	1.94	0.07	1.88	0.07	1.90	0.07	0.20	0.37	0.44	0.86	0.00	0.01
PC(16:0/16:0)	5.54	0.05	5.27	0.05	5.38	0.05	0.00	0.00	0.00	0.03	0.00	0.01
PC(16:0/18:1)	8.73	0.05	8.53	0.05	8.66	0.05	0.00	0.00	0.21	0.59	0.00	0.01
PC(16:0e/18:1(9Z))	3.92	0.05	3.79	0.05	3.89	0.05	0.00	0.01	0.32	0.73	0.00	0.02
PC(18:0p/18:1(9Z))	3.06	0.08	2.64	0.08	3.00	0.07	0.00	0.00	0.36	0.76	0.00	0.02
PC(18:0p/22:6)	2.28	0.06	2.01	0.06	2.24	0.06	0.00	0.00	0.58	0.94	0.00	0.03
PC(30:0)	4.34	0.08	4.17	0.08	4.32	0.09	0.05	0.14	0.89	0.99	0.00	0.03
PC(31:0)	2.28	0.10	2.10	0.10	2.25	0.11	0.10	0.24	0.77	0.96	0.00	0.03
PC(32:1)	5.87	0.07	5.69	0.07	5.84	0.08	0.02	0.07	0.71	0.96	0.01	0.04
PC(32:2)	3.26	0.09	3.33	0.09	3.28	0.09	0.34	0.52	0.78	0.96	0.01	0.04
PC(33:0)	2.31	0.10	2.19	0.10	2.41	0.10	0.18	0.35	0.22	0.61	0.01	0.04
PC(33:1)	3.95	0.06	3.86	0.06	3.98	0.07	0.13	0.28	0.42	0.83	0.01	0.05
PC(34:2)	9.02	0.05	8.93	0.05	8.96	0.05	0.03	0.08	0.17	0.53	0.01	0.05
PC(34:3)	5.20	0.07	5.23	0.07	5.23	0.08	0.71	0.82	0.71	0.96	0.01	0.05
PC(35:1)	4.58	0.05	4.52	0.05	4.64	0.06	0.20	0.37	0.20	0.59	0.01	0.05
PC(35:2)	5.06	0.06	5.08	0.06	5.14	0.06	0.64	0.76	0.05	0.31	0.01	0.05

PC(35:3)	3.22	0.12	3.21	0.12	3.34	0.11	0.98	0.98	0.35	0.76	0.01	0.07
PC(35:4)	2.68	0.07	2.78	0.07	2.72	0.06	0.03	0.08	0.29	0.70	0.01	0.07
PC(36:1)	6.74	0.05	6.43	0.05	6.65	0.06	0.00	0.00	0.18	0.55	0.01	0.07
PC(36:2)	8.48	0.05	8.32	0.05	8.44	0.06	0.00	0.01	0.47	0.87	0.01	0.07
PC(36:2)#	3.52	0.07	3.55	0.07	3.54	0.08	0.62	0.75	0.75	0.96	0.01	0.07
PC(36:3)	7.64	0.10	7.55	0.10	7.59	0.11	0.34	0.52	0.61	0.95	0.02	0.07
PC(36:3)#	7.66	0.08	7.63	0.08	7.68	0.08	0.73	0.83	0.78	0.96	0.02	0.08
PC(36:4)	7.16	0.03	7.09	0.03	7.13	0.03	0.02	0.06	0.42	0.83	0.02	0.09
PC(36:4)/(18:3/18:1)	4.01	0.06	3.80	0.06	4.02	0.07	0.00	0.00	0.79	0.96	0.02	0.09
PC(36:4)#	5.21	0.07	5.26	0.07	5.20	0.08	0.41	0.58	0.94	1.00	0.02	0.09
PC(36:4)_2	8.88	0.04	8.85	0.04	8.86	0.03	0.42	0.58	0.36	0.76	0.02	0.09
PC(36:4)_3	4.76	0.14	4.63	0.14	4.66	0.15	0.52	0.67	0.60	0.95	0.02	0.09
PC(36:5)	5.24	0.29	4.83	0.29	4.41	0.27	0.31	0.48	0.04	0.27	0.02	0.09
PC(36:5)#	6.12	0.06	6.03	0.06	5.95	0.07	0.24	0.41	0.05	0.31	0.02	0.09
PC(37:2)	2.67	0.09	2.68	0.09	2.75	0.09	0.90	0.94	0.34	0.74	0.03	0.10
PC(37:3)	3.54	0.08	3.65	0.08	3.64	0.09	0.10	0.24	0.27	0.68	0.03	0.10
PC(37:3)/(18:3/19:0)	3.46	0.05	3.20	0.05	3.35	0.06	0.00	0.00	0.13	0.48	0.03	0.11
PC(37:4)	4.29	0.12	4.49	0.12	4.47	0.12	0.02	0.07	0.12	0.46	0.03	0.11
PC(37:5)	2.37	0.13	2.10	0.13	2.28	0.14	0.13	0.28	0.57	0.94	0.03	0.11
PC(38:1)	2.32	0.06	2.03	0.06	2.15	0.07	0.00	0.01	0.04	0.28	0.03	0.11
PC(38:2)/(14:1/24:1)	4.02	0.11	3.60	0.11	3.88	0.09	0.01	0.03	0.14	0.51	0.03	0.11
PC(38:3)	6.31	0.07	6.23	0.07	6.32	0.07	0.33	0.52	0.94	1.00	0.03	0.11
PC(38:4)	7.66	0.06	7.64	0.06	7.66	0.06	0.63	0.75	0.96	1.00	0.04	0.14

PC(38:4)#	5.34	0.08	5.34	0.08	5.40	0.08	0.98	0.98	0.53	0.94	0.04	0.14
PC(38:5)	7.29	0.06	7.26	0.06	7.29	0.05	0.57	0.70	0.92	1.00	0.04	0.14
PC(38:6)	7.07	0.06	6.89	0.06	6.87	0.06	0.00	0.01	0.00	0.01	0.04	0.14
PC(38:6)#	4.68	0.05	4.69	0.05	4.66	0.05	0.90	0.94	0.63	0.96	0.05	0.15
PC(39:0)/(13:0/26:0)	2.71	0.08	2.60	0.08	2.68	0.08	0.11	0.24	0.72	0.96	0.05	0.15
PC(39:6)	4.00	0.09	3.89	0.09	3.86	0.10	0.12	0.27	0.08	0.40	0.05	0.15
PC(40:4)	3.09	0.07	2.98	0.07	3.19	0.07	0.13	0.28	0.11	0.45	0.05	0.15
PC(40:5)	4.90	0.06	4.84	0.06	4.90	0.06	0.34	0.52	0.93	1.00	0.05	0.15
PC(40:5)#	3.17	0.10	2.99	0.10	3.21	0.10	0.17	0.34	0.78	0.96	0.05	0.15
PC(40:6)	6.07	0.07	5.86	0.07	5.90	0.06	0.00	0.00	0.01	0.09	0.05	0.15
PC(40:6)#	4.25	0.05	4.22	0.05	4.23	0.05	0.61	0.74	0.66	0.96	0.06	0.17
PC(40:7)	4.30	0.07	4.24	0.07	4.19	0.06	0.25	0.43	0.05	0.31	0.06	0.17
PC(40:8)	2.17	0.08	1.95	0.08	1.97	0.09	0.00	0.00	0.03	0.27	0.06	0.18
PC(42:8)/(20:3/22:5)	2.61	0.13	2.36	0.13	2.34	0.14	0.18	0.35	0.10	0.44	0.06	0.18
PC(42:8)/(20:3/22:5)#	2.65	0.07	2.47	0.07	2.47	0.08	0.07	0.18	0.08	0.40	0.07	0.18
PC(44:5)/(22:2/22:3)	3.08	0.05	3.11	0.05	3.11	0.05	0.69	0.80	0.69	0.96	0.08	0.20
PC(O-32:0)	3.26	0.04	3.10	0.04	3.20	0.05	0.00	0.00	0.23	0.62	0.08	0.20
PC(O-32:1)	3.03	0.06	2.80	0.06	2.99	0.06	0.00	0.00	0.34	0.74	0.08	0.21
PC(O-34:2)	4.12	0.07	3.53	0.07	4.20	0.07	0.00	0.00	0.24	0.62	0.08	0.21
PC(O-34:3)	4.54	0.06	4.24	0.06	4.52	0.07	0.00	0.00	0.78	0.96	0.08	0.21
PC(O-34:3)NH4adduct	5.54	0.04	5.44	0.04	5.48	0.04	0.01	0.03	0.08	0.40	0.09	0.21
PC(O-36:3)	2.88	0.14	2.34	0.14	2.90	0.12	0.00	0.00	0.85	0.98	0.08	0.21
PC(O-36:3)#	3.33	0.14	2.89	0.14	3.61	0.12	0.00	0.01	0.05	0.31	0.09	0.22

PC(O-36:4)	5.26	0.05	4.88	0.05	5.29	0.04	0.00	0.00	0.46	0.87	0.09	0.22
PC(O-36:5)	4.95	0.06	4.64	0.06	4.93	0.06	0.00	0.00	0.69	0.96	0.10	0.23
PC(O-38:4)	2.63	0.09	2.34	0.09	2.63	0.10	0.04	0.11	0.98	1.00	0.10	0.24
PC(O-38:4)#	4.15	0.05	3.95	0.05	4.16	0.06	0.00	0.01	0.78	0.96	0.11	0.25
PC(O-38:4)#	3.42	0.12	2.90	0.12	3.17	0.14	0.01	0.02	0.08	0.40	0.13	0.29
PC(O-38:5)	5.09	0.04	4.91	0.04	5.09	0.04	0.00	0.00	0.97	1.00	0.13	0.30
PC(O-38:5)#	3.46	0.07	3.11	0.07	3.48	0.06	0.00	0.00	0.70	0.96	0.13	0.30
PC(O-38:6)	4.50	0.05	4.11	0.05	4.40	0.04	0.00	0.00	0.03	0.25	0.13	0.30
PC(O-38:6)#	2.25	0.11	1.64	0.11	1.97	0.11	0.00	0.00	0.03	0.25	0.13	0.30
PC(O-40:5)	2.92	0.06	2.80	0.06	2.92	0.06	0.14	0.28	1.00	1.00	0.13	0.30
PC(O-40:5)#	2.67	0.09	2.44	0.09	2.29	0.08	0.03	0.08	0.00	0.01	0.14	0.30
PC(O-42:5)/(o-22:1/20:4)	2.67	0.08	2.67	0.08	2.73	0.08	0.97	0.98	0.61	0.95	0.14	0.31
PC(P-18:0/22:6)	3.95	0.07	3.56	0.07	3.75	0.06	0.00	0.00	0.01	0.09	0.14	0.31
PE(16:0/18:1)	3.11	0.09	2.95	0.09	2.93	0.10	0.09	0.22	0.11	0.45	0.15	0.32
PE(34:2)	2.95	0.09	2.88	0.09	2.87	0.10	0.45	0.62	0.41	0.82	0.16	0.33
PE(36:4)	4.01	0.07	3.80	0.07	3.86	0.07	0.00	0.00	0.00	0.06	0.16	0.33
PE(38:4)	5.32	0.07	4.93	0.07	4.97	0.08	0.00	0.00	0.00	0.01	0.16	0.34
PE(38:6)	4.17	0.09	4.00	0.09	3.97	0.09	0.01	0.03	0.01	0.09	0.17	0.34
PE(O-38:5)orPE(P-38:4)	5.11	0.06	4.39	0.06	4.83	0.05	0.00	0.00	0.00	0.01	0.18	0.35
PE(P-18:0/22:6)	4.21	0.06	3.62	0.06	3.94	0.05	0.00	0.00	0.00	0.01	0.19	0.37
PG(O-39:0)/(O-20:0/19:0)	5.49	0.05	5.33	0.05	5.42	0.05	0.00	0.00	0.15	0.51	0.19	0.37
PG(O-41:0)/(O-20:0/21:0)	6.07	0.05	5.85	0.05	5.96	0.05	0.00	0.00	0.07	0.40	0.19	0.37
PI(18:0/20:4)	7.59	0.05	7.31	0.05	7.39	0.05	0.00	0.00	0.00	0.03	0.19	0.37

PI(38:7)	7.36	0.05	7.41	0.05	7.43	0.05	0.42	0.58	0.21	0.59	0.20	0.39
PI(44:4)	5.32	0.08	5.16	0.08	5.21	0.07	0.15	0.30	0.31	0.72	0.21	0.39
SM(37:1)	3.04	0.07	3.00	0.07	3.07	0.07	0.48	0.63	0.63	0.96	0.21	0.39
SM(39:2)	2.23	0.07	2.17	0.07	2.11	0.09	0.28	0.46	0.16	0.53	0.21	0.39
SM(40:2)	5.70	0.04	5.58	0.04	5.65	0.04	0.00	0.02	0.11	0.45	0.22	0.40
SM(d16:1/18:1)orSM(d18:2/16:0)	5.04	0.04	4.92	0.04	4.97	0.04	0.00	0.00	0.01	0.14	0.22	0.41
SM(d18:0/16:0)	3.75	0.05	3.60	0.05	3.67	0.06	0.00	0.01	0.16	0.53	0.23	0.41
SM(d18:1/12:0)	1.33	0.08	1.34	0.08	1.33	0.07	0.86	0.91	0.99	1.00	0.23	0.41
SM(d18:1/24:0)	5.30	0.07	5.20	0.07	5.23	0.07	0.28	0.46	0.40	0.81	0.24	0.43
SM(d18:2/14:0)	1.94	0.07	1.91	0.07	1.84	0.06	0.63	0.75	0.09	0.43	0.25	0.44
SM(d32:1)	4.77	0.05	4.70	0.05	4.69	0.05	0.06	0.16	0.04	0.28	0.26	0.45
SM(d33:1)	4.21	0.05	4.10	0.05	4.18	0.05	0.00	0.01	0.18	0.55	0.26	0.45
SM(d34:1)	7.43	0.04	7.25	0.04	7.31	0.04	0.00	0.00	0.00	0.01	0.27	0.47
SM(d35:1)	2.83	0.02	2.87	0.02	2.86	0.02	0.12	0.27	0.33	0.74	0.27	0.47
SM(d36:0)	1.84	0.14	1.80	0.14	1.81	0.15	0.77	0.86	0.86	0.98	0.27	0.47
SM(d36:1)	5.57	0.04	5.44	0.04	5.50	0.04	0.00	0.02	0.08	0.40	0.28	0.48
SM(d36:2)	5.00	0.04	4.87	0.04	4.95	0.04	0.00	0.02	0.17	0.53	0.29	0.49
SM(d38:2)	4.40	0.05	4.29	0.05	4.33	0.05	0.01	0.03	0.03	0.25	0.30	0.50
SM(d39:1)	4.12	0.06	4.12	0.06	4.13	0.06	0.91	0.95	0.83	0.97	0.30	0.50
SM(d40:1)	5.67	0.04	5.50	0.04	5.54	0.04	0.00	0.00	0.00	0.05	0.32	0.52
SM(d41:1)	4.77	0.05	4.74	0.05	4.74	0.05	0.54	0.68	0.56	0.94	0.32	0.52
SM(d41:2)	4.86	0.04	4.81	0.04	4.84	0.05	0.16	0.32	0.75	0.96	0.32	0.52

SM(d42:2)	6.49	0.04	6.34	0.04	6.40	0.03	0.00	0.00	0.01	0.12	0.33	0.53
SM(d42:3)	5.88	0.04	5.75	0.04	5.82	0.04	0.00	0.00	0.03	0.25	0.34	0.54
TG(14:0/16:0/18:1)	6.35	0.14	6.18	0.14	6.38	0.14	0.22	0.39	0.77	0.96	0.36	0.55
TG(14:0/18:1/18:1)	7.75	0.08	7.74	0.08	7.75	0.07	0.84	0.91	0.95	1.00	0.35	0.55
TG(14:0/18:2/18:2)	5.32	0.16	5.53	0.16	5.24	0.15	0.23	0.40	0.60	0.95	0.36	0.55
TG(16:0/16:0/12:0)	2.75	0.18	2.49	0.18	2.70	0.19	0.19	0.36	0.80	0.96	0.35	0.55
TG(16:0/16:0/16:0)	4.31	0.18	3.75	0.18	4.09	0.20	0.00	0.01	0.31	0.72	0.36	0.55
TG(16:0/18:0/18:1)	6.45	0.14	6.02	0.14	6.48	0.13	0.01	0.02	0.78	0.96	0.38	0.58
TG(16:0/18:2/18:2)	7.93	0.08	8.11	0.08	7.90	0.08	0.10	0.24	0.67	0.96	0.39	0.60
TG(16:0/18:2/18:3)	6.05	0.14	6.31	0.14	6.01	0.13	0.13	0.28	0.79	0.96	0.41	0.62
TG(16:0/18:2/18:3)#	5.71	0.17	5.76	0.17	5.57	0.15	0.78	0.87	0.46	0.87	0.41	0.62
TG(16:0/18:2/22:6)	5.03	0.14	5.02	0.14	4.74	0.12	0.93	0.96	0.04	0.28	0.43	0.63
TG(16:0/22:5/18:1)orTG(20:4/18:1/18:1)	6.99	0.08	7.03	0.08	6.94	0.07	0.68	0.80	0.56	0.94	0.44	0.65
TG(16:1/18:1/12:0)	4.66	0.18	4.51	0.18	4.63	0.18	0.39	0.56	0.86	0.98	0.44	0.65
TG(18:0/18:1/20:4)	6.73	0.07	6.66	0.07	6.63	0.07	0.31	0.48	0.10	0.44	0.46	0.66
TG(18:1/12:0/18:1)orTG(18:2/16:0/14:0)	6.35	0.14	6.35	0.14	6.35	0.13	0.97	0.98	1.00	1.00	0.46	0.66
TG(18:1/18:1/16:0)	9.14	0.04	9.11	0.04	9.10	0.04	0.48	0.63	0.09	0.41	0.47	0.67
TG(18:1/18:1/18:1)	8.40	0.06	8.44	0.06	8.35	0.05	0.46	0.62	0.27	0.68	0.47	0.67
TG(18:1/18:2/18:2)	7.26	0.11	7.30	0.11	7.29	0.09	0.79	0.88	0.78	0.96	0.49	0.68
TG(18:2/18:1/16:0)	8.97	0.05	9.04	0.05	8.91	0.04	0.21	0.38	0.13	0.47	0.49	0.68
TG(18:2/18:1/18:1)	8.20	0.06	8.36	0.06	8.18	0.05	0.03	0.09	0.65	0.96	0.48	0.68

TG(18:2/18:1/18:1)#	6.31	0.12	6.44	0.12	6.46	0.11	0.27	0.45	0.30	0.72	0.49	0.69
TG(18:2/18:2/18:2)orTG(18:3/18:2/18:1)	6.12	0.18	6.38	0.18	6.07	0.18	0.26	0.44	0.79	0.96	0.50	0.69
TG(18:2/22:5/16:0)	7.02	0.11	6.92	0.11	6.73	0.10	0.37	0.55	0.00	0.05	0.51	0.69
TG(46:0)/(14:0/16:0/16:0)	3.88	0.17	3.57	0.17	3.94	0.17	0.07	0.18	0.67	0.96	0.50	0.69
TG(46:2)/(16:1/14:0/16:1)	3.45	0.21	3.52	0.21	3.38	0.21	0.76	0.85	0.73	0.96	0.54	0.73
TG(47:0)	1.94	0.16	1.88	0.16	2.37	0.17	0.76	0.85	0.03	0.25	0.55	0.74
TG(47:1)	3.44	0.16	3.38	0.16	3.52	0.17	0.69	0.80	0.60	0.95	0.56	0.74
TG(48:0)	3.41	0.14	3.21	0.14	3.44	0.16	0.13	0.28	0.87	0.98	0.56	0.74
TG(48:1)	5.13	0.11	5.01	0.11	5.17	0.11	0.22	0.40	0.64	0.96	0.56	0.74
TG(48:2)/(14:1/16:1/18:0)	4.41	0.11	4.41	0.11	4.42	0.11	0.98	0.98	0.84	0.97	0.57	0.75
TG(48:3)	4.73	0.19	4.91	0.19	4.66	0.19	0.37	0.55	0.67	0.96	0.61	0.78
TG(48:4)	4.03	0.07	3.98	0.07	3.98	0.07	0.46	0.62	0.47	0.87	0.61	0.78
TG(49:0)	3.22	0.15	3.05	0.15	3.32	0.17	0.30	0.48	0.55	0.94	0.60	0.78
TG(49:0)#	3.03	0.18	3.00	0.18	3.29	0.18	0.86	0.91	0.21	0.59	0.62	0.79
TG(49:1)	5.19	0.12	5.09	0.12	5.24	0.12	0.36	0.55	0.60	0.95	0.62	0.80
TG(49:2)	5.12	0.11	5.13	0.11	5.14	0.12	0.94	0.97	0.83	0.97	0.64	0.81
TG(49:3)/(15:0/18:2/16:1)	3.28	0.16	3.25	0.16	3.20	0.16	0.85	0.91	0.54	0.94	0.64	0.81
TG(50:0)	4.13	0.07	4.19	0.07	4.03	0.07	0.40	0.57	0.12	0.46	0.66	0.81
TG(50:0)#	4.23	0.16	3.73	0.16	4.40	0.16	0.00	0.02	0.30	0.72	0.65	0.81
TG(50:1)	7.67	0.12	7.38	0.12	7.65	0.11	0.01	0.04	0.82	0.97	0.66	0.81
TG(50:1)#	3.82	0.10	3.94	0.10	3.77	0.09	0.31	0.48	0.64	0.96	0.66	0.81
TG(50:2)	6.40	0.06	6.37	0.06	6.38	0.06	0.67	0.79	0.79	0.96	0.65	0.81

TG(50:3)	7.34	0.10	7.48	0.10	7.33	0.09	0.17	0.33	0.98	1.00	0.66	0.81
TG(50:3)#	5.67	0.08	5.79	0.08	5.68	0.08	0.16	0.32	0.84	0.97	0.69	0.82
TG(50:5)	2.64	0.18	2.52	0.18	2.33	0.17	0.53	0.67	0.16	0.53	0.68	0.82
TG(51:1)	5.18	0.13	5.04	0.13	5.33	0.12	0.37	0.55	0.25	0.64	0.68	0.82
TG(51:2)	6.49	0.09	6.51	0.09	6.49	0.10	0.72	0.82	1.00	1.00	0.69	0.82
TG(51:2)#	3.41	0.14	3.37	0.14	3.49	0.14	0.81	0.89	0.50	0.91	0.69	0.82
TG(51:2)_2	5.63	0.09	5.65	0.09	5.63	0.09	0.83	0.91	0.91	1.00	0.73	0.85
TG(51:3)	5.83	0.09	5.97	0.09	5.83	0.10	0.16	0.32	0.92	1.00	0.73	0.85
TG(51:4)	4.28	0.14	4.40	0.14	4.19	0.15	0.46	0.62	0.47	0.87	0.72	0.85
TG(52:0)	1.47	0.15	1.56	0.15	1.93	0.14	0.58	0.71	0.01	0.11	0.73	0.85
TG(52:2)	7.44	0.04	7.41	0.04	7.39	0.04	0.48	0.63	0.11	0.45	0.79	0.91
TG(52:3)	7.27	0.05	7.37	0.05	7.26	0.05	0.17	0.33	0.82	0.97	0.81	0.92
TG(52:4)	6.60	0.06	6.73	0.06	6.56	0.06	0.11	0.24	0.53	0.94	0.84	0.93
TG(52:5)	5.70	0.08	5.94	0.08	5.73	0.08	0.04	0.10	0.81	0.97	0.83	0.93
TG(52:6)	3.86	0.20	4.07	0.20	3.85	0.18	0.42	0.58	0.96	1.00	0.84	0.93
TG(53:2)	5.56	0.12	5.47	0.12	5.61	0.11	0.38	0.55	0.57	0.94	0.84	0.93
TG(53:3)	5.10	0.07	5.23	0.07	5.10	0.08	0.09	0.23	0.99	1.00	0.83	0.93
TG(53:3)	6.13	0.08	6.25	0.08	6.13	0.08	0.10	0.24	1.00	1.00	0.84	0.93
TG(53:4)	5.25	0.10	5.44	0.10	5.30	0.09	0.11	0.24	0.55	0.94	0.82	0.93
TG(53:5)	3.72	0.17	3.59	0.17	3.68	0.15	0.41	0.58	0.78	0.96	0.84	0.93
TG(54:1)	3.68	0.16	3.01	0.16	3.71	0.16	0.00	0.00	0.84	0.97	0.85	0.93
TG(54:1)/TG(16:0/22:1/16:0)	3.22	0.14	2.77	0.14	3.23	0.15	0.01	0.03	0.96	1.00	0.87	0.94
TG(54:3)	6.80	0.05	6.87	0.05	6.77	0.04	0.23	0.40	0.41	0.82	0.87	0.94

TG(54:4)	6.62	0.05	6.78	0.05	6.58	0.05	0.02	0.06	0.39	0.80	0.89	0.96
TG(54:5)	5.98	0.09	6.30	0.09	5.96	0.07	0.01	0.02	0.75	0.96	0.89	0.96
TG(54:6)	6.62	0.13	6.53	0.13	6.51	0.12	0.53	0.67	0.39	0.81	0.91	0.96
TG(54:7)	4.79	0.19	4.80	0.19	4.43	0.18	0.94	0.97	0.12	0.46	0.91	0.96
TG(54:7)#	4.77	0.16	4.81	0.16	4.45	0.17	0.84	0.91	0.10	0.44	0.90	0.96
TG(55:1)	3.12	0.11	2.81	0.11	3.16	0.10	0.02	0.06	0.70	0.96	0.91	0.96
TG(55:5)	4.01	0.12	4.11	0.12	4.14	0.11	0.45	0.61	0.24	0.63	0.93	0.97
TG(56:3)	5.65	0.08	5.60	0.08	5.57	0.07	0.54	0.68	0.17	0.53	0.96	0.97
TG(56:3)/TG(18:0/20:3/18:0)	4.59	0.08	4.51	0.08	4.53	0.07	0.37	0.55	0.32	0.74	0.94	0.97
TG(56:4)	5.55	0.12	5.62	0.12	5.55	0.11	0.61	0.74	1.00	1.00	0.94	0.97
TG(56:4)/TG(20:1/16:0/20:3)	4.16	0.12	4.27	0.12	4.19	0.12	0.53	0.67	0.90	1.00	0.95	0.97
TG(56:5)	5.80	0.10	5.75	0.10	5.85	0.11	0.71	0.81	0.67	0.96	0.95	0.97
TG(56:6)	6.04	0.06	6.10	0.06	6.00	0.05	0.46	0.62	0.53	0.94	0.98	0.99
TG(58:6)	4.79	0.09	4.93	0.09	4.86	0.09	0.29	0.47	0.59	0.95	0.98	0.99
TG(58:9)	4.32	0.15	4.38	0.15	4.04	0.13	0.69	0.80	0.07	0.40	0.99	1.00
TG(60:7)	2.45	0.11	2.26	0.11	2.07	0.10	0.24	0.41	0.01	0.13	1.00	1.00

The least square mean and standard error of plasma lipid levels were obtained by mixed modelling. Cer: ceramide; CE: cholesteryl ester; DAG: diacylglyceride; HexCer: Hexosylceramide; Hs-CRP: high- sensitive c-reactive protein; LacCer: lactosylceramide; LPC: lyso-phosphatidylcholine; O-PC: ether phosphatidylcholine; PC: phosphatidylcholine; PE: phosphatidylethanolamine; PG: phosphatidylglycerol, PI: phosphatidylinositol; SM: sphingomyelin; TG: Triacylglycerol. # Lipids with same fatty acyl chains (Cx:y) were presented in the dataset, where x represents the number of carbon atoms and y the number of double bonds of the fatty acyl chain.

Table S4. Cardiovascular risk factor levels after four weeks of a vegetarian diet compared to a meat diet.

Clinical parameters	Pre VD	Post VD	Pre MD	Post MD	Post VD vs Post MD#	$P^{\#}$
Oxidized LDL-C, U/L	41.4 (37.2–45.5)	37.5 (33.8–40.7)**	41.8 (37.7–46.0)	40.0 (35.9–44.2)	-2.73 (-4.9– -0.6)	0.02
TC, mmol/L	3.5 (3.2–3.7)	3.2 (2.9–3.4)***	3.5 (3.4–3.7)	3.3 (3.1–3.6)*	-0.1 (-0.2–0.03)	0.01
LDL-C, mmol/L	1.6 (1.4–1.8)	1.4 (1.3–1.5)***	1.7 (1.5–1.8)	1.5 (1.4–1.7)*	-0.1 (-0.2–0.02)	0.02
Weight, kg	84.1 (80.1–88.2)	83.7 (79.5–87.9)	84.7 (80.5–88.9)	84.4 (80.1–88.6)	-0.7 (-1.1–0.2)	0.008
BMI, kg/m²	27.4 (26.4–28.5)	27.3 (26.2–28.4)	27.6 (26.5–28.7)	27.5 (26.4–28.6)	-0.2 (-0.36–0.06)	0.009

Data are presented as mean (95% CI) or as geometric mean [95 % CI]. Within-group change in *P*-value was calculated with paired t-test. *$P < 0.05$,**$P < 0.01$,***$P < 0.001$. #Differences in clinical parameters between vegetarian diet (VD) and meat diet (MD) were examined using linear mixed-effects models adjusted for sequence of randomisation and period of interventions. BMI, body mass index; LDL-C, low-density lipoprotein cholesterol; MD, meat diet; TC, total cholesterol, TG; triacylglycerol, VD: vegetarian diet.

.

Table S5. Loadings of lipids that differed between vegetarian diet and meat diet derived from principle component analysis.

	Component 1	Component 2	Component 3	Component 4
TG(56:6)	0.48	0.84		
TG(16:0/18:2/22:6)	0.25	0.83		-0.19
TG(54:5)	0.21	0.82		0.25
TG(50:1)	0.49	0.80		
TG(16:0/18:2/18:2)	0.50	0.79		-0.10
TG(54:4)	0.30	0.79	-0.23	0.21
TG(52:4)	0.55	0.79		
TG(18:2/18:1/18:1)	0.33	0.78	-0.13	0.19
TG(58:9)	0.22	0.77		-0.48
TG(53:4)	0.54	0.77	0.25	
TG(16:0/18:2/18:3)	0.45	0.74	0.29	-0.17
TG(52:3)	0.56	0.74		
TG (53:3)	0.55	0.72		0.27
TG(53:3)	0.52	0.72	0.22	
TG(18:2/18:1/16:0)	0.56	0.70	-0.15	
DG (36:3)	0.31	0.69	-0.12	
TG(18:2/22:5/16:0)	0.43	0.69	0.21	-0.27
TG(52:5)	0.48	0.69	0.21	0.31
TG(18:2/18:2/18:2)	0.34	0.68	0.24	0.20
TG(50:0)	0.56	0.62		
TG(54:7)	0.33	0.58	0.19	-0.45
TG(51:3)	0.67	0.56	0.31	
TG(50:3)	0.62	0.50	0.43	
TG(50:3)	0.63	0.50	0.46	-0.12
TG(14:0/18:2/18:2)	0.51	0.47	0.56	
SM(d36:2)	0.77	-0.11	-0.30	-0.24
PC(16:0e/18:1(9Z))	0.88	-0.11	-0.38	
SM(d33:1)	0.85	-0.13	-0.20	-0.11
HexCer(d18:1/22:0)	0.51	-0.15	-0.21	0.46
PC(P-18:0/22:6)	0.78	-0.15	-0.16	
PC (36:4) /(18:3/18:1)	0.21	-0.16	-0.25	
PC(O-38:5)	0.81	-0.16	-0.40	
PC(O-38:4)	0.81	-0.16	-0.24	-0.28
PE(O-38:5) or PE(P-38:4)	0.84	-0.16	-0.20	

PG (O-39:0)	0.81	-0.17	-0.35	
TG(48:1)	0.49	-0.17	0.81	
PC(36:2)	0.88	-0.18		0.13
PC(O-34:2)	0.71	-0.19		0.27
PE(P-18:0/22:6)	0.82	-0.19	-0.10	
PC(O-34:3)	0.91	-0.20	-0.14	
PC(O-36:5)	0.77	-0.23	-0.37	-0.15
CE(18:0)	0.82	-0.23	-0.14	
Cer(d18:1/16:0)	0.47	-0.23	0.20	0.38
PC(O-38:4)	0.65	-0.23	-0.53	-0.22
PC (37:3)	0.77	-0.24	-0.16	
TG (55:1)	0.74	-0.26	0.48	
PC(O-32:1)	0.75	-0.27	-0.45	
TG(47:0)	0.20	-0.27	0.69	-0.25
PC(40:4)	0.67	-0.28		-0.27
PC(36:1)	0.85	-0.30		
PC(O-38:5)	0.78	-0.30	-0.36	-0.19
PC(35:1)	0.84	-0.30		-0.23
TG (16:0/22:1/16:0)	0.55	-0.31	0.54	0.24
PC(O-38:6)	0.83	-0.31	-0.23	
PC(O-36:3)	0.49	-0.32		
TG(51:1)	0.48	-0.32	0.66	
TG(50:1)	0.62	-0.33	0.62	0.15
TG(54:1)	0.59	-0.33	0.44	0.31
TG (14:0/16:0/16:0)	0.21	-0.34	0.80	-0.14
PC(O-36:4)	0.83	-0.35	-0.25	
PC(O-38:6)	0.38	-0.38	0.21	0.70
PC(33:1)	0.69	-0.38	0.37	-0.34
PC(16:0/16:0)	0.79	-0.39		-0.17
PC(32:1)	0.71	-0.39	0.25	-0.32
PC(33:0)	0.57	-0.39	0.25	-0.33
PC(16:0/18:1)	0.75	-0.41	0.13	-0.15
TG(16:0/18:0/18:1)	0.54	-0.42	0.53	0.30
LPC(16:0e)		-0.45		-0.35
PC(18:0p/22:6)	0.55	-0.45	-0.19	0.29
TG(50:0)	0.34	-0.59	0.65	0.19
PC(18:0/18:1)	0.75		-0.11	0.34

PC(O-32:0)	0.73		-0.45	0.21
PC(O-36:3)	0.61		-0.22	0.23
PG (O-41:0)	0.78		-0.44	0.11

Cer, ceramide; LPC, lyso-phosphatidylcholine; PC, phosphatidylcholine; PE, phosphatidylethanolamine; TG, Triacylglycerol.

Table S6. Association of lipid levels with coronary artery disease burden.

	**The total number of stenotic lesions**			The Sullivan extent score		
	**β-coefficient**	**SE**	***P***	β-coefficient	SE	*P*
CE(16:0)	0.02	1.96	0.99	-0.12	0.65	0.86
CE(16:1)	-0.10	2.15	0.96	0.06	0.75	0.93
CE(18:0)	-1.07	3.84	0.78	-0.74	1.26	0.56
CE(18:1)	1.13	2.12	0.60	0.59	0.69	0.40
CE(18:2)	-0.67	2.14	0.76	0.04	0.71	0.95
CE(20:4)	0.70	2.29	0.76	0.01	0.76	0.98
CE(20:5)	1.85	2.18	0.41	0.06	0.73	0.94
Cer(d18:1/22:0)	1.34	2.77	0.63	1.06	0.89	0.25
Cer(d18:1/16:0)	0.82	1.98	0.68	0.69	0.64	0.29
Cer(d18:1/22:0)	3.76	2.45	0.14	1.92	0.73	0.02
Cer(d18:1/23:0)	0.14	2.33	0.95	0.09	0.78	0.91
Cer(d18:1/24:0)	-1.56	2.84	0.59	-0.38	0.95	0.69
Cer(d18:1/24:1)	0.67	2.36	0.78	0.73	0.77	0.35
Cer(d18:1/25:0)	-1.39	2.04	0.50	-0.57	0.73	0.44
Cer(d42:1)	-2.36	2.97	0.44	-0.52	1.00	0.61
DG(36:3)	-1.94	1.86	0.31	-0.63	0.63	0.33
HexCer(d18:1/22:0)	0.80	2.15	0.71	-0.22	0.71	0.76
HexCer(d18:1/24:0)	0.54	2.31	0.82	0.13	0.78	0.87
LacCer(d18:1/16:0)	-1.36	2.75	0.63	0.51	0.95	0.60
LPC(16:0)	3.25	2.35	0.18	1.09	0.88	0.23
LPC(16:0e)	2.44	2.30	0.30	0.57	0.77	0.47
LPC(16:0p)	3.65	1.86	0.07	1.38	0.63	0.04
LPC(18:0)	4.99	3.06	0.12	1.62	1.03	0.13
LPC(18:1)	5.54	2.76	0.06	2.17	0.87	0.02
LysoPC(15:0)	2.61	2.21	0.25	0.38	0.78	0.63
LysoPC(14:0)	2.88	2.26	0.22	0.54	0.94	0.57
PC(O-42:5)	-3.31	2.02	0.12	-1.06	0.67	0.13
PC(36:4)	1.23	1.91	0.53	0.29	0.64	0.65

PC(37:3)	2.62	2.52	0.31	1.47	0.79	0.08
PC(38:1)	4.81	2.27	0.05	1.68	0.74	0.04
PC(38:2)	4.28	2.28	0.08	1.15	0.78	0.15
PC(39:0)	0.66	2.02	0.75	0.19	0.68	0.78
PC(42:8)	1.19	2.16	0.59	0.59	0.71	0.42
PC(42:8)	3.41	2.40	0.17	1.46	0.76	0.07
PC(44:5)	-2.10	2.28	0.37	-0.05	0.78	0.95
PC(16:0/16:0)	3.14	2.01	0.13	1.03	0.67	0.14
PC(16:0/18:1)	3.59	2.20	0.12	1.33	0.74	0.09
PC(16:0e/18:1(9Z))	-1.17	2.18	0.60	0.06	0.77	0.94
PC(18:0p/18:1(9Z))	-1.44	2.24	0.53	-0.24	0.78	0.77
PC(18:0p/22:6)	1.35	2.13	0.53	-0.10	0.74	0.90
PC(30:0)	3.26	2.21	0.16	0.75	0.81	0.37
PC(31:0)	2.18	2.51	0.40	0.70	0.86	0.43
PC(32:1)	1.94	2.17	0.38	0.85	0.77	0.28
PC(32:2)	0.04	2.64	0.99	0.08	0.93	0.93
PC(33:0)	1.94	2.05	0.36	0.43	0.70	0.55
PC(33:1)	2.89	2.07	0.18	0.86	0.72	0.25
PC(34:2)	-0.43	2.57	0.87	0.44	0.89	0.63
PC(34:3)	1.78	2.18	0.42	0.25	0.74	0.74
PC(35:1)	3.60	2.15	0.11	1.11	0.72	0.14
PC(35:2)	1.56	3.00	0.61	1.05	0.99	0.30
PC(35:3)	0.61	2.59	0.82	-0.55	0.85	0.53
PC(35:4)	1.54	1.96	0.44	0.27	0.67	0.70
PC(36:1)	6.94	2.54	0.01	2.25	0.84	0.01
PC(36:2)	0.70	3.69	0.85	1.17	1.20	0.34
PC(36:2)#	-0.41	2.33	0.86	0.15	0.80	0.86
PC(36:3)	0.30	2.39	0.90	0.88	0.76	0.26
PC(36:3)#	2.43	2.11	0.26	0.72	0.74	0.34
PC(36:4)	3.76	1.82	0.05	1.03	0.63	0.12
PC(36:4)#	0.79	2.67	0.77	0.46	0.88	0.61
PC(36:4)#	0.63	2.00	0.76	0.34	0.66	0.61
PC(36:4)_3	2.07	2.25	0.37	0.58	0.77	0.46
PC(36:5)	-1.38	1.96	0.49	0.39	0.68	0.57
PC(36:5)#	0.43	2.39	0.86	0.05	0.80	0.95
PC(37:2)	1.34	2.32	0.57	0.74	0.75	0.34
PC(37:3)	4.00	2.64	0.15	1.30	0.88	0.16

PC(37:4)	0.16	2.02	0.94	0.25	0.67	0.71
PC(37:5)	3.69	2.14	0.10	1.30	0.71	0.08
PC(38:3)	3.27	2.42	0.19	1.11	0.80	0.18
PC(38:4)	0.04	1.98	0.98	0.14	0.66	0.83
PC(38:4)#	3.62	2.47	0.16	1.41	0.83	0.10
PC(38:5)	0.21	2.30	0.93	0.32	0.76	0.68
PC(38:6)	3.10	1.96	0.13	0.63	0.68	0.37
PC(38:6)#	0.87	3.24	0.79	0.73	1.07	0.50
PC(39:6)	6.14	1.94	0.01	1.53	0.71	0.04
PC(40:4)	3.13	1.85	0.11	1.39	0.58	0.03
PC(40:5)	2.04	1.95	0.31	0.93	0.67	0.18
PC(40:5)#	3.74	2.11	0.09	1.14	0.74	0.14
PC(40:6)	3.48	2.02	0.10	0.63	0.71	0.38
PC(40:6)#	3.21	2.12	0.15	1.21	0.72	0.11
PC(40:7)	4.44	2.11	0.05	1.36	0.71	0.07
PC(40:8)	4.00	2.25	0.09	1.47	0.73	0.06
PC(O-32:0)	0.57	2.27	0.80	0.09	0.75	0.91
PC(O-32:1)	0.58	2.24	0.80	0.43	0.78	0.59
PC(O-34:2)	-0.02	2.09	0.99	-0.35	0.70	0.62
PC(O-34:3)	1.01	2.10	0.64	0.08	0.71	0.91
PC(O-34:3)NH4adduct	2.88	2.86	0.33	0.48	0.96	0.62
PC(O-36:3)	-1.09	1.91	0.57	0.06	0.66	0.93
PC(O-36:3)#	1.39	2.33	0.56	-0.14	0.78	0.86
PC(O-36:4)	1.28	2.21	0.57	0.28	0.73	0.70
PC(O-36:5)	1.71	2.06	0.42	0.32	0.69	0.65
PC(O-38:4)	-0.57	2.22	0.80	-0.23	0.74	0.76
PC(O-38:4)#	-0.14	2.22	0.95	0.30	0.75	0.70
PC(O-38:4)#	3.01	1.82	0.12	0.38	0.65	0.57
PC(O-38:5)	1.01	2.57	0.70	0.67	0.85	0.44
PC(O-38:5)#	1.13	2.09	0.60	0.48	0.70	0.50
PC(O-38:6)	2.02	2.57	0.44	0.69	0.85	0.43
PC(O-38:6)#	-1.98	2.11	0.36	-0.88	0.69	0.22
PC(O-40:5)	-1.98	2.20	0.38	-0.39	0.76	0.61
PC(O-40:5)#	-1.74	2.05	0.41	-0.52	0.68	0.45
PC(P-18:0/22:6)	1.18	2.34	0.62	0.32	0.80	0.69
PE(16:0/18:1)	2.86	1.73	0.12	1.66	0.67	0.02
PE(34:2)	1.14	2.01	0.58	0.62	0.71	0.39

PE(36:4)	2.29	1.80	0.22	0.95	0.60	0.13
PE(38:4)	3.27	1.72	0.07	1.32	0.54	0.02
PE(38:6)	1.70	1.85	0.37	0.53	0.62	0.40
PE(O-38:5)orPE(P-38:4)	3.49	1.67	0.05	1.42	0.51	0.01
PE(P-18:0/22:6)	3.09	2.03	0.15	0.86	0.68	0.22
PG(O-39:0)	2.92	3.28	0.38	0.82	1.09	0.46
PG(O-41:0)	1.89	2.83	0.51	0.83	0.96	0.40
PI(38:7)	2.17	2.03	0.30	1.23	0.64	0.07
PI(44:4)	-1.05	2.64	0.70	-0.22	0.93	0.82
PI(18:0/20:4)	1.43	2.29	0.54	0.69	0.76	0.38
SM(37:1)	0.67	2.13	0.76	0.08	0.71	0.91
SM(40:2)	0.79	2.85	0.78	0.50	0.94	0.60
SM(d39:1)	0.77	2.28	0.74	0.08	0.76	0.92
SM(d42:3)	-0.11	2.46	0.97	0.58	0.81	0.48
SM(39:2)	0.18	2.26	0.94	0.02	0.75	0.98
SM(d16:1/18:1)orSM(d18:2 /16:0)	-2.99	2.72	0.29	0.20	0.93	0.84
SM(d18:0/16:0)	1.54	2.79	0.59	0.64	0.92	0.49
SM(d18:1/12:0)	2.18	2.27	0.35	1.44	1.05	0.18
SM(d18:1/24:0)	2.82	2.73	0.31	0.75	0.93	0.43
SM(d18:2/14:0)	-1.60	2.52	0.53	-0.14	0.84	0.87
SM(d32:1)	-1.24	3.09	0.69	0.07	1.08	0.95
SM(d33:1)	0.32	2.66	0.91	0.49	0.88	0.59
SM(d34:1)	-1.76	2.94	0.56	0.39	0.98	0.70
SM(d35:1)	0.73	1.84	0.69	0.10	0.62	0.88
SM(d36:0)	0.26	2.55	0.92	0.41	0.84	0.63
SM(d36:1)	0.93	2.50	0.71	0.30	0.83	0.72
SM(d36:2)	-0.85	2.73	0.76	0.31	0.91	0.73
SM(d38:2)	1.56	2.48	0.54	0.83	0.82	0.33
SM(d40:1)	1.36	2.93	0.65	0.83	0.97	0.40
SM(d41:1)	-0.01	2.37	1.00	-0.21	0.79	0.79
SM(d41:2)	0.57	2.37	0.81	0.28	0.78	0.73
SM(d42:2)	-0.16	2.56	0.95	0.47	0.87	0.59
TG(46:0)	2.44	1.81	0.19	0.49	0.68	0.49
TG(46:2)	2.13	1.91	0.28	0.32	0.71	0.65
TG(48:2)	2.55	1.84	0.18	0.54	0.75	0.48
TG(48:4)	1.48	2.43	0.55	0.08	0.81	0.92

TG(49:3)	1.21	1.96	0.54	0.06	0.70	0.93
TG(52:0)	0.57	2.05	0.78	0.63	0.69	0.38
TG(53:3)	1.55	1.94	0.43	0.04	0.68	0.96
TG(54:1)	0.37	1.99	0.85	-0.07	0.68	0.92
TG(55:1)	2.28	1.88	0.24	0.41	0.68	0.56
TG(56:3)	2.09	1.82	0.26	0.51	0.61	0.41
TG(56:4)	-1.89	2.15	0.39	0.59	0.72	0.43
TG(14:0/16:0/18:1)	2.75	1.80	0.14	0.57	0.72	0.44
TG(14:0/18:1/18:1)	2.38	1.81	0.20	0.47	0.72	0.52
TG(14:0/18:2/18:2)	1.01	1.93	0.61	-0.06	0.68	0.93
TG(16:0/16:0/12:0)	2.74	1.89	0.16	0.52	0.69	0.46
TG(16:0/16:0/16:0)	1.80	1.86	0.35	0.44	0.70	0.53
TG(16:0/18:0/18:1)	1.21	2.00	0.55	0.22	0.74	0.77
TG(16:0/18:2/18:2)	0.01	1.93	1.00	-0.32	0.65	0.63
TG(16:0/18:2/18:3)	0.16	1.92	0.94	-0.29	0.65	0.66
TG(16:0/18:2/18:3)#	0.24	2.02	0.91	-0.28	0.69	0.69
TG(16:0/18:2/22:6)	-1.16	1.93	0.56	-0.65	0.64	0.32
TG(16:0/22:5/18:1)orTG(20:4/18:1/18:1)	0.68	2.01	0.74	-0.28	0.67	0.68
TG(16:1/18:1/12:0)	2.91	1.81	0.12	0.56	0.72	0.45
TG(18:0/18:1/20:4)	1.30	2.01	0.52	0.10	0.69	0.89
TG(18:1/12:0/18:1)orTG(18:2/16:0/14:0)	2.53	1.86	0.19	0.40	0.74	0.60
TG(18:1/18:1/16:0)	1.83	1.89	0.35	0.19	0.70	0.79
TG(18:1/18:1/18:1)	1.38	1.87	0.47	0.25	0.62	0.69
TG(18:1/18:2/18:2)	3.55	1.95	0.09	0.69	0.71	0.34
TG(18:2/18:1/16:0)	-0.29	2.01	0.89	-0.43	0.70	0.55
TG(18:2/18:1/18:1)	0.55	1.87	0.77	-0.02	0.62	0.98
TG(18:2/18:1/18:1)#	3.17	1.84	0.10	0.69	0.69	0.33
TG(18:2/18:2/18:2)orTG(18:3/18:2/18:1)	-0.46	1.93	0.81	-0.59	0.64	0.37
TG(18:2/22:5/16:0)	-1.00	1.90	0.60	-0.55	0.63	0.39
TG(47:0)	-0.33	1.95	0.87	-0.39	0.64	0.55
TG(47:1)	2.61	1.83	0.17	0.37	0.66	0.58
TG(48:0)	2.47	1.82	0.19	0.53	0.67	0.44
TG(48:1)	2.78	1.80	0.14	0.59	0.72	0.42
TG(48:3)	1.89	1.90	0.33	0.17	0.71	0.82
TG(49:0)	2.24	1.78	0.22	0.53	0.66	0.43

TG(49:0)#	2.29	1.82	0.22	0.58	0.63	0.37
TG(49:1)	1.83	1.87	0.34	0.15	0.71	0.83
TG(49:2)	1.82	1.93	0.36	0.22	0.72	0.76
TG(50:0)	-0.83	2.00	0.68	-0.55	0.68	0.43
TG(50:0)#	2.15	1.83	0.25	0.49	0.69	0.49
TG(50:1)	2.02	1.86	0.29	0.38	0.72	0.60
TG(50:1)#	-0.17	1.95	0.93	-0.32	0.67	0.63
TG(50:2)	1.90	1.84	0.31	0.38	0.72	0.61
TG(50:3)	1.44	1.91	0.46	0.14	0.70	0.85
TG(50:3)#	1.41	1.86	0.46	0.11	0.67	0.87
TG(50:5)	0.00	1.99	1.00	-0.41	0.67	0.55
TG(51:1)	2.85	1.76	0.12	0.47	0.67	0.49
TG(51:2)	1.91	1.92	0.33	0.17	0.71	0.81
TG(51:2)#	1.88	1.86	0.33	0.11	0.69	0.88
TG(51:2)_2	1.60	1.97	0.43	0.12	0.72	0.87
TG(51:3)	1.09	1.98	0.59	-0.02	0.70	0.97
TG(51:4)	1.61	1.96	0.42	0.12	0.67	0.86
TG(52:2)	1.74	1.88	0.37	0.22	0.69	0.75
TG(52:3)	1.77	1.91	0.37	0.11	0.66	0.87
TG(52:4)	-0.15	1.90	0.94	-0.39	0.65	0.56
TG(52:5)	1.02	1.89	0.60	-0.09	0.64	0.89
TG(52:6)	-1.40	2.01	0.49	-0.43	0.70	0.55
TG(53:2)	2.20	1.92	0.27	0.27	0.71	0.71
TG(53:3)	1.41	1.92	0.47	-0.02	0.68	0.98
TG(53:4)	1.22	1.88	0.52	-0.02	0.64	0.98
TG(53:5)	1.18	1.94	0.55	0.36	0.66	0.59
TG(54:1)	2.67	2.38	0.28	0.24	0.84	0.78
TG(54:3)	1.32	1.86	0.49	0.20	0.62	0.75
TG(54:4)	0.14	1.90	0.94	-0.03	0.63	0.96
TG(54:5)	-0.16	1.91	0.93	-0.16	0.64	0.81
TG(54:6)	-0.74	2.01	0.72	-0.70	0.67	0.30
TG(54:7)	-2.37	1.92	0.23	-0.71	0.66	0.29
TG(54:7)#	-0.85	2.00	0.68	-0.67	0.66	0.33
TG(55:5)	0.60	1.94	0.76	-0.09	0.65	0.89
TG(56:3)	1.88	1.79	0.31	0.41	0.60	0.51
TG(56:4)	0.57	1.85	0.76	0.06	0.62	0.93
TG(56:5)	3.61	1.70	0.05	0.98	0.59	0.11

TG(56:6)	-0.06	2.19	0.98	-0.50	0.72	0.50
TG(58:6)	3.15	2.18	0.17	0.38	0.76	0.62
TG(58:9)	-1.18	1.94	0.55	-0.59	0.63	0.36
TG(60:7)	0.42	2.16	0.85	-0.05	0.72	0.95

Associations of baseline lipid levels with the total number of stenotic lesions and the Sullivan extent score were assessed using linear regression adjusted for age, sex, BMI, and total cholesterol. The β-coefficient and standard error obtained from linear regression are presented. Cer, ceramide; CE, cholesteryl ester; DAG, diacylglycerides; HexCer, hexosylceramide; LacCer, lactosylceramide; LPC, lyso-phosphatidylcholine; NS, non-significant; O-PC, ether phosphatidylcholine; PC, phosphatidylcholine; PE, phosphatidylethanolamine; PG, phosphatidylglycerol; PI, phosphatidylinositol; SM: sphingomyelin; TAG, Triacylglycerol.

Table S7. Association of plasma lipid levels at baseline with coronary artery disease burden prior to percutanous coronary intervention.

Lipid species	β-coefficient ± SE	*P*	VD vs. baseline	MD vs. baseline	VD vs. MD
Association with the number of stenotic lesions					
PE(O-38:5)	1.42 ± 0.51	0.01	Decreased	Decreased	Decreased
PC(36:1)	2.25 ± 0.84	0.01	Decreased	NS	Decreased
Cer(d18:1/22:0)	1.92 ± 0.73	0.02	Decreased	NS	NS
LPC(18:1)	2.17 ± 0.87	0.02	NS	NS	NS
PE(16:0/18:1)	1.66 ± 0.67	0.02	NS	NS	NS
PE(38:4)	1.32 ± 0.54	0.02	Decreased	Decreased	NS
PC(40:4)	1.39 ± 0.58	0.03	NS	NS	Decreased
PC(38:1)	1.68 ± 0.74	0.04	Decreased	NS	NS
LPC(16:0)	1.38 ± 0.63	0.04	NS	NS	NS
PC(39:6)	1.53 ± 0.71	0.04	NS	NS	NS
Association with the Sullivan extent score					
PC(39:6)	6.14 ±1.94	0.01	NS	NS	NS
PC(36:1)	6.94 ± 2.54	0.01	Decreased	NS	Decreased
TG(56:5)	3.61 ±1.70	0.05	NS	NS	NS
PC (38:1)	4.81 ± 2.27	0.05	Decreased	NS	Decreased
PC(40:7)	4.44 ± 2.11	0.05	NS	NS	NS
PE(O-38:5)	3.49 ±1.67	0.05	Decreased	Decreased	Decreased

Associations of baseline lipids with the number of stenotic lesions and the Sullivan extent score were assessed using linear regression adjusted for age, sex, BMI, and total cholesterol. The β-coefficient and standard error obtained from linear regression are presented. Differences in lipid levels in VD and MD vs. baseline and between VD and MD assessed using generalized linear mixed models. NS: non-significant. Cer, ceramide; LPC, lyso-phosphatidylcholines; PC, phosphatidylcholine; PE, phosphatidylethanolamine;TG, Triacylglycerol.

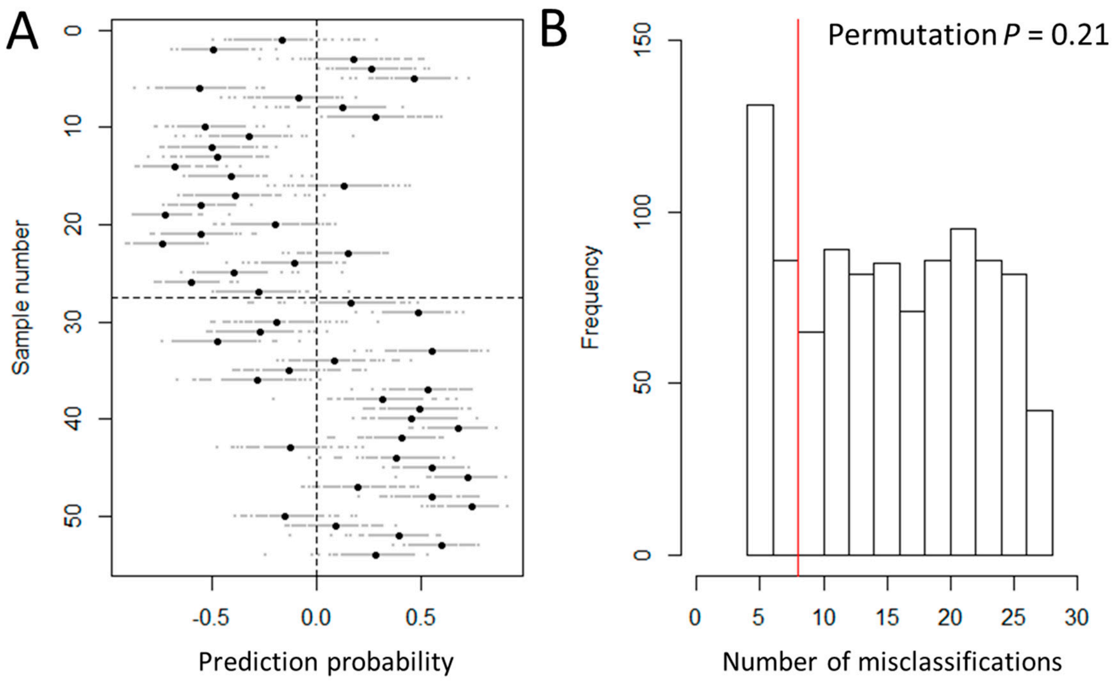

Figure S1. The model performance and permutation test of plasma lipid profiles at baseline and after wash-out period using the random forest modeling based multilevel data analysis. A:Classification performance of multivariate modelling.B: Results from permutation test.

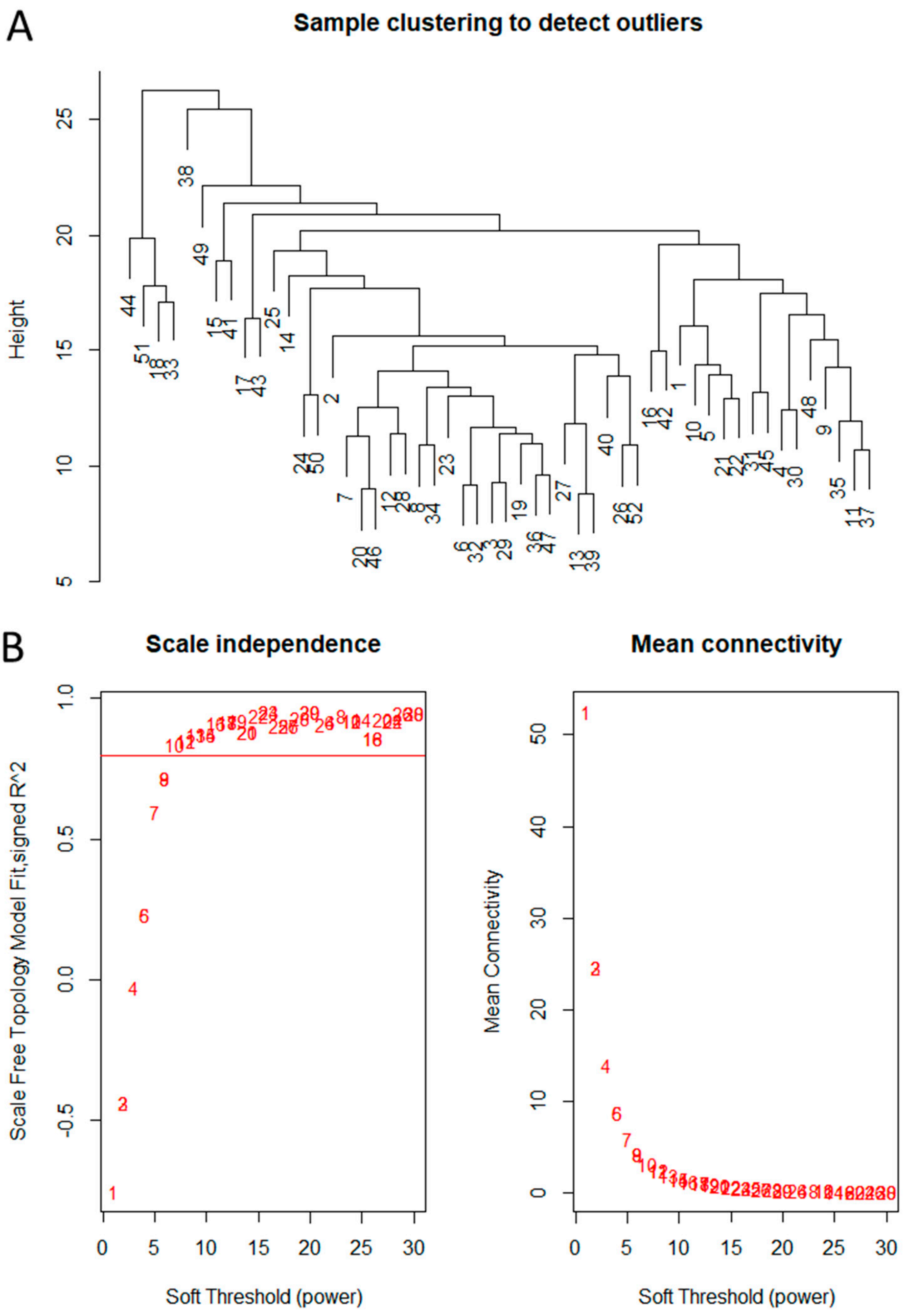

Figure S2. Data checking and power determined by Scale-Free Topology Criterion for the weighted gene correlation network analysis.

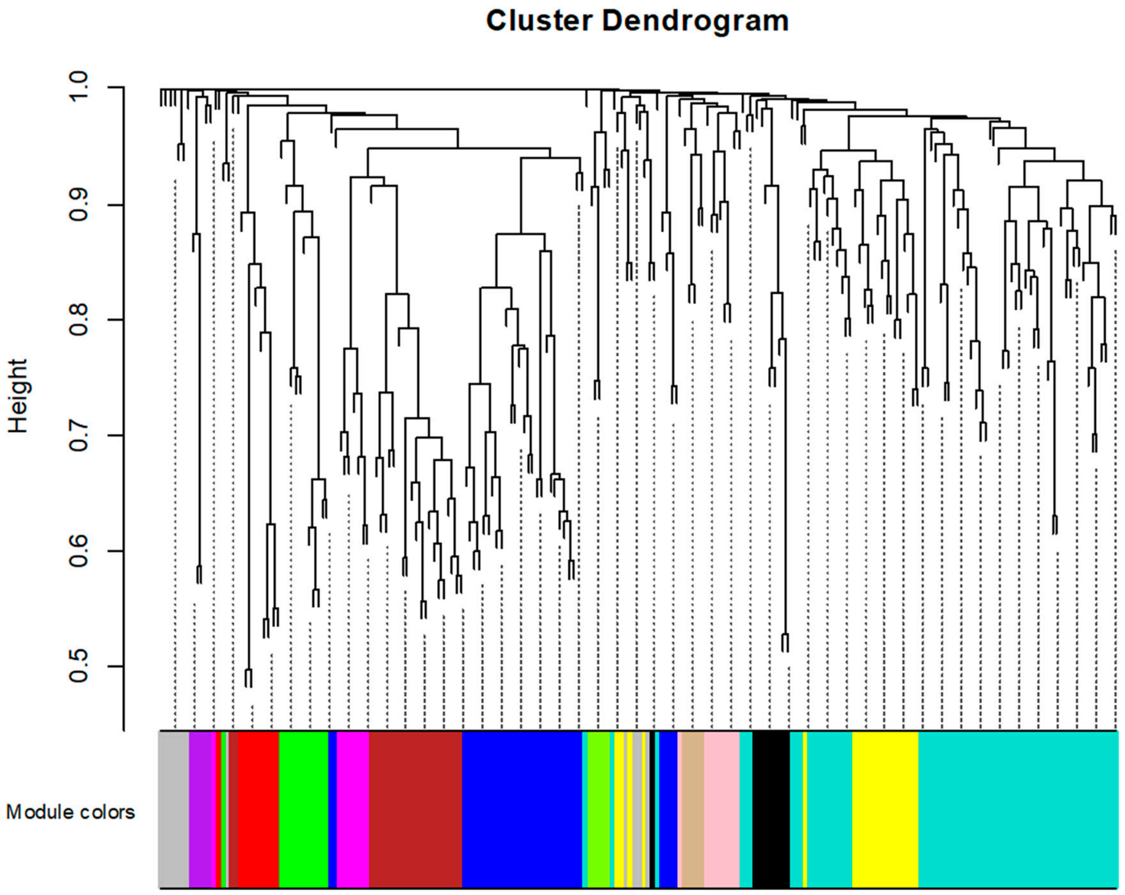

Figure S3. Dendrogram created from a dynamic tree-cutting algorithm for the weighted gene correlation network analysis.

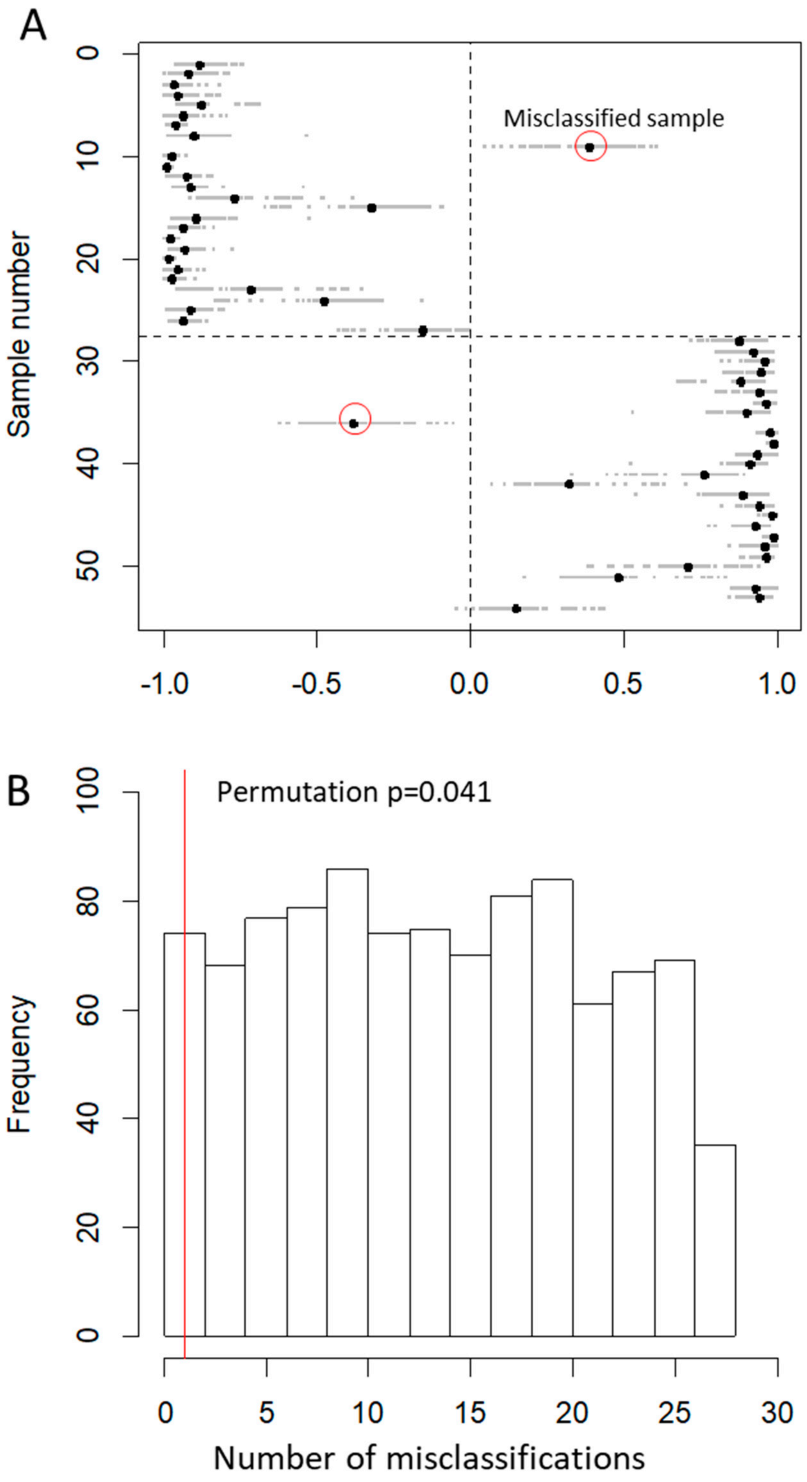

Figure S4. The effect of diet on plasma lipid profiles assessed by multilevel random forest modelling. The model performance (A) and permutation test (B).

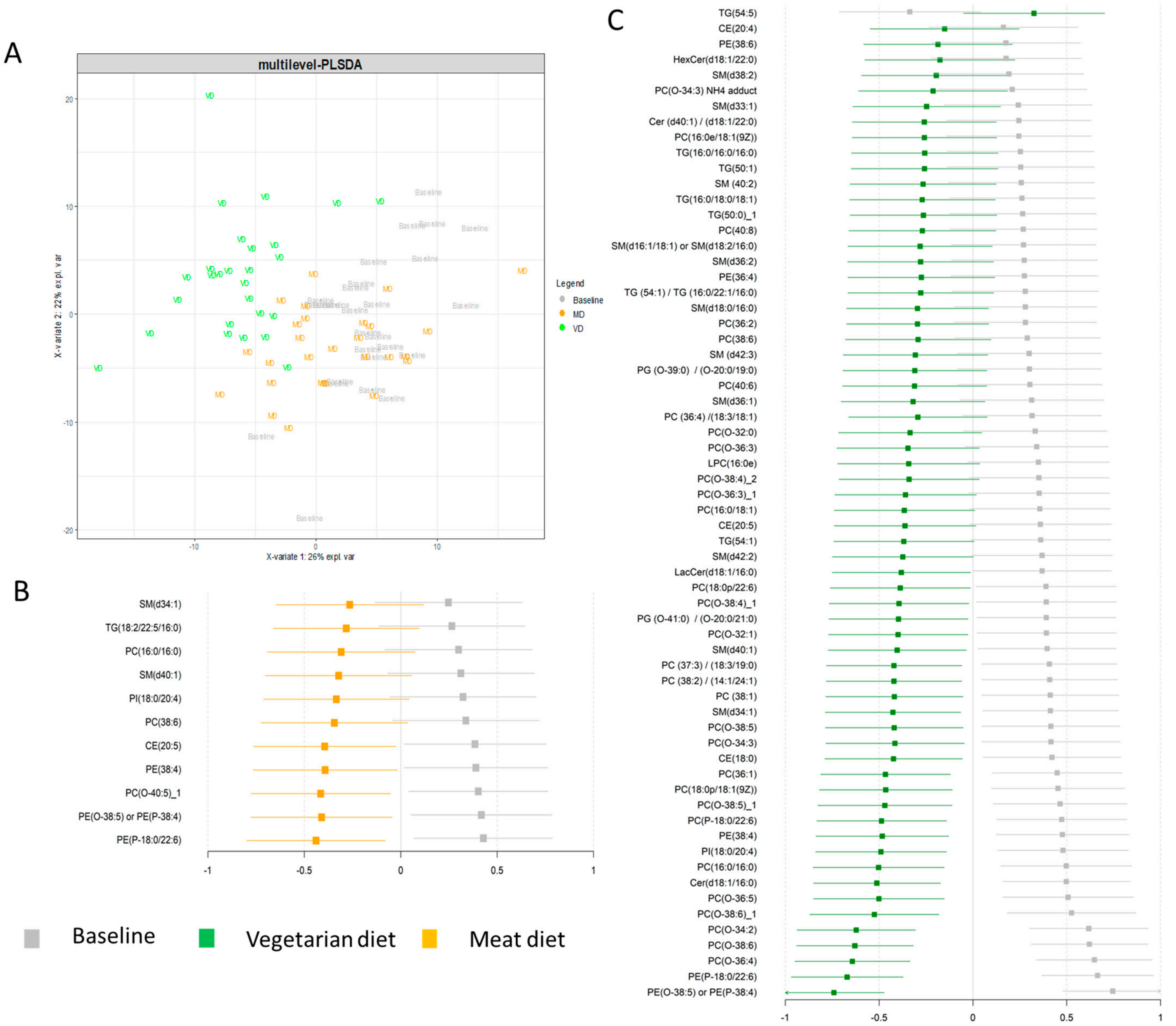
A
multilevel-PLSDA
X-variate 1: 26% expl. var
X-variate 2: 22% expl. var
Legend
Baseline
MD
VD
B
SM(d34:1)
TG(18:2/22:5/16:0)
PC(16:0/16:0)
SM(d40:1)
PI(18:0/20:4)
PC(38:6)
CE(20:5)
PE(38:4)
PC(O-40:5)_1
PE(O-38:5) or PE(P-38:4)
PE(P-18:0/22:6)
C
TG(54:5)
CE(20:4)
PE(38:6)
HexCer(d18:1/22:0)
SM(d38:2)
PC(O-34:3) NH4 adduct
SM(d33:1)
Cer (d40:1) / (d18:1/22:0)
PC(16:0e/18:1(9Z))
TG(16:0/16:0/16:0)
TG(50:1)
SM (40:2)
TG(16:0/18:0/18:1)
TG(50:0)_1
PC(40:8)
SM(d16:1/18:1) or SM(d18:2/16:0)
SM(d36:2)
PE(36:4)
TG (54:1) / TG (16:0/22:1/16:0)
SM(d18:0/16:0)
PC(36:2)
PC(38:6)
SM (d42:3)
PG (O-39:0) / (O-20:0/19:0)
PC(40:6)
SM(d36:1)
PC (36:4) /(18:3/18:1)
PC(O-32:0)
PC(O-36:3)
LPC(16:0e)
PC(O-38:4)_2
PC(O-36:3)_1
PC(16:0/18:1)
CE(20:5)
TG(54:1)
SM(d42:2)
LacCer(d18:1/16:0)
PC(18:0p/22:6)
PC(O-38:4)_1
PG (O-41:0) / (O-20:0/21:0)
PC(O-32:1)
SM(d40:1)
PC (37:3) / (18:3/19:0)
PC (38:2) / (14:1/24:1)
PC (38:1)
SM(d34:1)
PC(O-38:5)
PC(O-34:3)
CE(18:0)
PC(36:1)
PC(18:0p/18:1(9Z))
PC(O-38:5)_1
PC(P-18:0/22:6)
PE(38:4)
PI(18:0/20:4)
PC(16:0/16:0)
Cer(d18:1/16:0)
PC(O-36:5)
PC(O-38:6)_1
PC(O-34:2)
PC(O-38:6)
PC(O-36:4)
PE(P-18:0/22:6)
PE(O-38:5) or PE(P-38:4)
Baseline
Vegetarian diet
Meat diet

Figure S5. The effect of diet on plasma lipid profiles. (A) Score plot of the multilevel partial least squares discrimination analysis modelling. Differences in plasma lipids among vegetarian diet, meat diet, and baseline. (B) Differences from baseline in individual lipids with vegetarian diet and with meat diet (C) Assessment by generalized linear mixed modelling. Standardized values of least-squares means and 95% confidence interval illustrated for comparison. Cer: ceramide; CE: cholesteryl ester; DAG: diacylglyceride; HexCer: Hexosylceramide; Hs-CRP: high- sensitive c-reactive protein; LacCer: lactosylceramide; LPC: lyso-phosphatidylcholine; O-PC: ether phosphatidylcholine; PC: phosphatidylcholine; PE: phosphatidylethanolamine; PG: phosphatidylglycerol, PI: phosphatidylinositol; SM: sphingomyelin; TG: Triacylglycerol. # Lipids with same fatty acyl chains (Cx:y) were presented in the dataset, where x represents the number of carbon atoms and y the number of double bonds of the fatty acyl chain.
